# The role of MNK1-mTORC1 pathway in modulating macrophage responses to *Vibrio vulnificus* infection

**DOI:** 10.1128/spectrum.03340-23

**Published:** 2024-07-09

**Authors:** Yong-Liang Lou, Dan-Li Xie, Xian-Hui Huang, Meng-Meng Zheng, Na Chen, Ji-Ru Xu

**Affiliations:** 1 Department of Immunology and Pathogenic Biology, School of Medicine, Xi'an Jiaotong University, Xi'an, Shanxi, China; 2 The School of Laboratory Medicine and Life Sciences, Wenzhou Medical University, Wenzhou, Zhejiang, China; 3 Key Laboratory of Laboratory Medicine, Ministry of Education of China, Wenzhou Medical University, Wenzhou, Zhejiang, China; 4 Wenzhou Key Laboratory of Sanitary Microbiology, Wenzhou, Zhejiang, China; 5 Scientific Research Center, Wenzhou Medical University, Wenzhou, Zhejiang, China; 6 Department of Laboratory Medicine, The First People’s Hospital of Linping District, Hangzhou, Zhejiang, China; McGill University, Quebec, Canada

**Keywords:** *Vibrio vulnificus*, MNK, mTOR, macrophage, phagocytosis, inflammation, bactericidal activity

## Abstract

*Vibrio vulnificus* (*Vv*) is known to cause life-threatening infections, particularly septicemia. These patients often exhibit elevated levels of pro-inflammatory cytokines. While it is established that mitogen-activated protein kinase (MAPK)-interacting kinase (MNK) contributes to the production of pro-inflammatory cytokines, the role of MNK in macrophages during *Vv* infection remains unclear. In this study, we investigate the impact of MNK on macrophages. We demonstrate that the inhibition of MNK in J774A.1 cells, when treated with lipopolysaccharide or *Vv*, resulted in decreased production of tumor necrosis factor alpha and interleukin-6, without affecting their transcription. Interestingly, treatment with MNK inhibitor CGP57380 led to enhanced phosphorylation of MNK1 but decreased phosphorylation of eIF4E. Moreover, MNK1 knockout cells exhibited an increased capacity for phagocytosis and clearance of *Vv*, with more acidic phagosomes than the parental cells. Notably, CGP57380 did not impact phagocytosis, bacterial clearance, or phagosome acidification in *Vv*-infected J774A.1 cells. Considering the reported association between MNK and mammalian target of rapamycin complex 1 (mTORC1) activation, we investigated the mTORC1 signaling in MNK1 knockout cells infected with *Vv*. Our results revealed that attenuation of the mTORC1 signaling in these cells and treatment with the mTORC1 inhibitor rapamycin significantly enhanced bacterial clearance in J774A.1 cells following *Vv* infection. In summary, our findings suggest that MNK promotes the *Vv*-induced cytokine production in J774A.1 cells without affecting their transcription levels. MNK1 appears to impair the phagocytosis, bacterial clearance, and phagosome acidification in *Vv*-infected J774A.1 cells through the MNK1-mTORC1 signaling pathway rather than the MNK1-eIF4E signaling pathway. Our findings highlight the importance of the MNK1-mTORC1 pathway in modulating macrophage responses to *Vv* infection.

**IMPORTANCE:**

Mitogen-activated protein kinase (MAPK)-interacting kinase (MNK) plays a role in promoting the production of tumor necrosis factor alpha and interleukin-6 in macrophages during *Vibrio vulnificus* (*Vv*) infection. Inhibition or knockout of MNK1 in J774A.1 cells resulted in reduced cytokine production without affecting their transcription levels. MNK1 also impairs phagocytosis, bacterial clearance, and phagosome acidification in *Vv*-infected cells through the MNK1-mammalian target of rapamycin complex 1 (mTORC1) signaling pathway. The findings highlight the importance of the MNK1-mTORC1 pathway in modulating macrophage responses to *Vv* infection.

## INTRODUCTION

*Vibrio vulnificus* (*Vv*) is a Gram-negative marine bacterium that can cause life-threatening human infections (1). Individuals with underlying medical conditions such as diabetes and chronic alcoholic liver disease are particularly susceptible to *Vv* infection (2). Interestingly, *Vv* infections have a relatively short incubation period, with symptoms typically appearing within 24 h. Moreover, the mortality rate for primary septicemia caused by *Vv* is over 50%, and patients often succumb to the infection within 72 h (3, 4). Given the acute nature of *Vv* infection, it is crucial for the innate immune cells, especially macrophages, to mount an effective response. Several virulence factors produced by *Vv*, such as lipopolysaccharide (LPS), VvpM, hemolysin, and multifunctional autoprocessing repeats-in-toxin (MARTX), have been shown to promote the production of pro-inflammatory factors by macrophages (5–7). Additionally, MARTX has been found to inhibit phagocytosis of *Vv*-infected macrophages (8, 9). However, the precise mechanisms underlying the regulation of *Vv*-mediated responses in macrophages remain unclear.

The mitogen-activated protein kinase (MAPK)-interacting protein kinases 1 and 2 (MNK1 and MNK2) have been identified as key regulators of mRNA translation by phosphorylating the translation initiation factor eIF4E (10). Studies have shown that MNK1 or MNK2 regulates genes involved in early, transient, and late immune responses to pathogens. Inhibiting MNK activity has been found to attenuate the production of pro-inflammatory factors such as tumor necrosis factor alpha (TNF-α), interleukin (IL)-6, and monocyte chemoattractant protein (MCP)-1, while enhancing the production of the anti-inflammatory cytokine IL-10 in macrophages treated with Toll-like receptor (TLR) agonists such as LPS, imiquimod, fibroblast-stimulating lipopeptide (FSL), and flagellin (11, 12). Furthermore, inhibition of MNK activity has decreased the secretion of CXCL8, CCL-3, and CCL4 in human neutrophils stimulated with LPS or TNF-α (13). Not only does MNK affect the production of cytokines and chemokines in innate immune cells, but it also regulates cytokine production in T cells. Inhibition of MNK activity has been found to reduce the production of interferon gamma (IFN-γ) and IL-4 in natural killer T cells stimulated with anti-CD3 monoclonal antibodies (14). In an experimental autoimmune encephalomyelitis model, the absence of MNKs resulted in attenuated production of IFN-γ and IL-17 by CD4 T cells (15). These findings highlight the important role of MNKs in modulating immune responses by regulating cytokine and chemokine production in innate immune cells and T cells.

The mammalian target of rapamycin (mTOR) is a protein that exists in two distinct complexes, mTOR complex 1 (mTORC1) and mTOR complex 2 (mTORC2). Recent research has highlighted the crucial role of mTORC1 and mTORC2 in regulating cytokine production in various innate immune cell populations (16). Inhibition of mTORC1 using tristetraprolin has enhanced bacterial clearance in macrophages (17). Moreover, the mTORC1 inhibitor rapamycin has been shown to restore the antimicrobial activity in HPS1 knockout HAP1 cells (18). Previous studies have demonstrated that MNK maintains mTORC1 activity and contributes to the signaling pathway associated with mTORC1 in T-cell activation (19). Recent studies have revealed that *Mycobacterium tuberculosis* deactivates the PI3K/AKT/mTORC1 and MNK regulatory pathways, inducing a pro-tissue destructive phenotype (20).

Our previous study demonstrated that *Vv* triggers a potent inflammatory response in macrophages by directly stimulating the production of various pro-inflammatory cytokines, including TNF-α, IL-6, and IL-1β (21). In this study, our focus was to investigate the contribution of MNK1 in promoting bacterial clearance within macrophages during *Vv* infection.

## RESULTS

### The inhibition of MNK1 in J774A.1 cells stimulated by LPS and *Vv* resulted in a reduction in protein levels of TNF-α and IL-6 without significantly impacting their transcription levels

**Fig 1:**
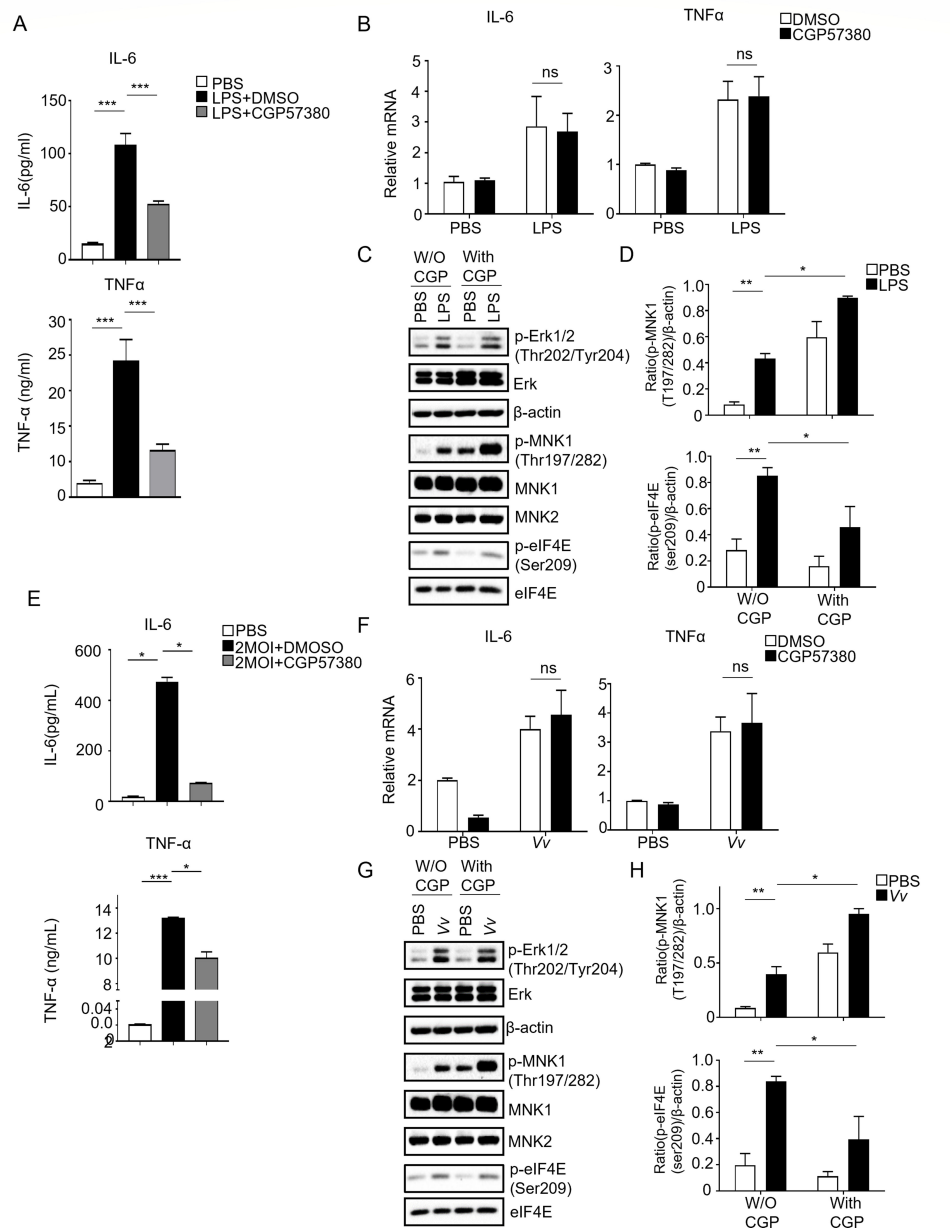
The production of IL-6 and TNF-α by J774A.1 cells treated with LPS or *Vv* was reduced by the MNK inhibitor CGP57380. (**A**) The levels of IL-6 and TNF-α were measured by ELISA in J774A.1 cells pretreated with 15-µM CGP57380 30 min before stimulated with 1-µg/mL LPS for 6 h. (**B**) The transcription of IL-6 and TNF-α was measured by RT-PCR in J774A.1 cells pretreated with 15-µM CGP57380 30 min before stimulated with 1-µg/mL LPS for 6 h. (**C**) Western blot determined the phosphorylation of MNK1, eIF4E, and ERK in J774A.1 cells pretreated with 15-µM CGP57380 30 min before stimulated with 1-µg/mL LPS for 1 h. (**D**) The bar figure shows the phosphorylation of MNK1 and eIF4E levels in J774A.1 cells pretreated with 15-µM CGP57380 30 min before stimulated with 1-µg/mL LPS for 1 h. (**E**) The levels of IL-6 and TNF-α were measured by ELISA in J774A.1 cells pretreated with 15-µM CGP57380 30 min before infected with 2 MOI *Vv* for 6 h. (**F**) The transcription of IL-6 and TNF-α was measured by RT-PCR in J774A.1 cells pretreated with 15-µM CGP57380 30 min before infected with 2 MOI *Vv* for 6 h. (**G**) Western blot determined the phosphorylation of MNK1, eIF4E, and ERK in J774A.1 cells pretreated with 15-µM CGP57380 30 min before infected with 2 MOI *Vv* for 1 h.(**H**) The bar figure shows the phosphorylation of MNK1 and eIF4E levels in J774A.1 cells pretreated with 15-µM CGP57380 30 min before infected with 2 MOI *Vv* for 1 h. *, *P* < 0.05; **, *P* < 0.01; ***, *P* < 0.001, determined by Student’s *t*-test.

Previous studies have demonstrated that the pharmacological inhibition of MNKs using CGP57380 decreases the production of pro-inflammatory cytokines, such as TNF-α and IL-6, in RAW264.7 cells treated with LPS (22). Furthermore, it has been observed that inhibiting MNK activity leads to a noticeable attenuation of eIF4E phosphorylation at Ser209. In our experiment using J774A.1 cells, we sought to investigate whether CGP57380 would have a similar effect. Our results indicate that pretreatment with CGP57380 before LPS stimulation for 6 h significantly reduced the expression levels of TNF-α and IL-6 in J774A.1 cells (Fig. 1A). There were no changes in the transcript levels of these cytokines (Fig. 1B). Additionally, the phosphorylation of eIF4E was dramatically decreased with CGP57380 treatment (Fig. 1C and D). Interestingly, we observed increased phosphorylation of MNK1, while the total protein levels of MNK1, MNK2, and their upstream protein Erk remained unaltered after LPS stimulation (Fig. 1C and D). This aligns with the findings by Boris V, who also noted that CGP57380 increased phosphorylation of MNK levels after LPS stimulation in bone marrow-derived macrophages (BMMϕ) (23). However, the underlying mechanism for this observation remained unknown in their research.

It is well-known that *Vv* can induce inflammation and cytokine production in activated macrophages and B cells (21, 24). Moreover, sera from *Vv* septicemic patients have been found to contain higher levels of pro-inflammatory cytokines, such as TNF-α and IL-6, compared to healthy individuals (25). Therefore, we aimed to investigate the role of MNK1 in producing pro-inflammatory cytokines by J774A.1 cells during *Vv* infection. To achieve this, we measured the expression levels of TNF-α and IL-6 in J774A.1 cells stimulated with *Vv* that had been pretreated with the MNK inhibitor CGP57380. Interestingly, we observed a decrease in the expression levels of TNF-α and IL-6 after 6 h of infection with 2 multiplicity of infection (MOI) *Vv* when pretreated with CGP57380 (Fig. 1E). However, the transcript levels of TNF-α and IL-6 showed minimal changes (Fig. 1F). Additionally, the phosphorylation of eIF4E was significantly reduced, while the phosphorylation of MNK1 was increased in response to *Vv* infection. Notably, the total protein levels of MNK1, MNK2, and their upstream protein Erk remained unchanged (Fig. 1G and H).

### MNK1-mediated activation of J774A.1 cells infected with *Vv* led to the induction of pro-inflammatory cytokine production

**Fig 2:**
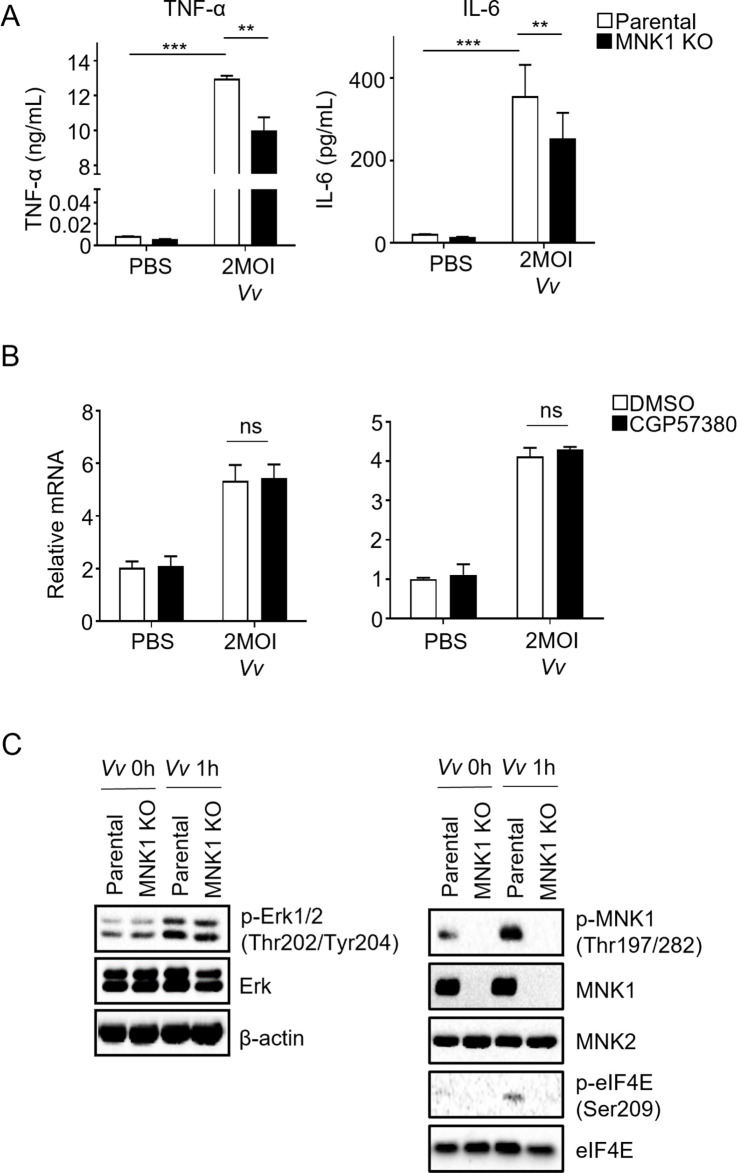
MNK1 promoted the production of TNF-α and IL-6 by J774A.1 cells in response to *Vv* infection, without inducing changes in transcription. (**A**) The levels of IL-6 and TNF-α were measured by ELISA in the supernatant from MNK1 knockout and parental cells after 2 MOI of *Vv* infection for 6 h. (**B**) The transcription of IL-6 and TNF-α was measured by RT-PCR in MNK1 knockout and parental cells after 2 MOI of *Vv* infection for 6 h. (**C**) Western blot determined the phosphorylation of MNK1, eIF4E, and ERK in MNK1 knockout and parental cells infected with 2 MOI *Vv* for 1 h. Data shown are representative of at least three experiments. *, *P* < 0.05; **, *P* < 0.01; ***, *P* < 0.001, determined by Student’s *t*-test.

Based on the findings mentioned above, it was observed that the MNK inhibitor CGP57380 could effectively inhibit the phosphorylation of eIF4E but not MNK1 phosphorylation when treated with *Vv* or LPS. This raised the question of whether the knockout of MNK1 would yield similar effects as the MNK inhibitor CGP57380. According to previous reports, MNK1 regulates eIF4E phosphorylation in response to external stimuli, while basal MNK2 activity is naturally high in cells and contributes to constitutive eIF4E phosphorylation levels (10). To further investigate the role of MNK1 in the biological functions of macrophages, we generated an MNK1^−/−^ J774A.1 cell line using CRISPR-Cas9 technology. Initially, we employed single-cell sorting via flow cytometry (FACS) to isolate J774A.1 cells containing the PX458-MNK1-green fluorescent protein (GFP) vector. Subsequently, cells that exhibited MNK1 protein knockout were selected (Fig. S1A), and their DNA was extracted to perform T7E1 assay and sequencing to confirm the homozygosity of the knockout cells (Fig. S1B and C). To investigate the impact of MNK1 knockout on pro-inflammatory cytokine production in J774A.1 cells during *Vv* infection, we compared the levels of TNF-α and IL-6 in parental and MNK1^−/−^ cells infected by *Vv*. Interestingly, we observed a decrease in the levels of TNF-α and IL-6 in MNK1^−/−^ cells after 6 h of infection with 2 MOI *Vv* (Fig. 2A). However, there was no significant difference in the transcription of TNF-α and IL-6 between the parental cells and MNK1^−/−^ cells (Fig. 2B). Additionally, eIF4E phosphorylation was reduced in MNK1^–/−^ cells following *Vv* infection (Fig. 2C). These findings indicate that the knockout of MNK1 produces a similar effect to that of the MNK inhibitor on TNF-α and IL-6 production and transcription in *Vv*-infected J774A.1 cells.

### The knockout of MNK1 in J774A.1 cells was found to enhance the phagocytosis and elimination of *Vv*

**Fig 3:**
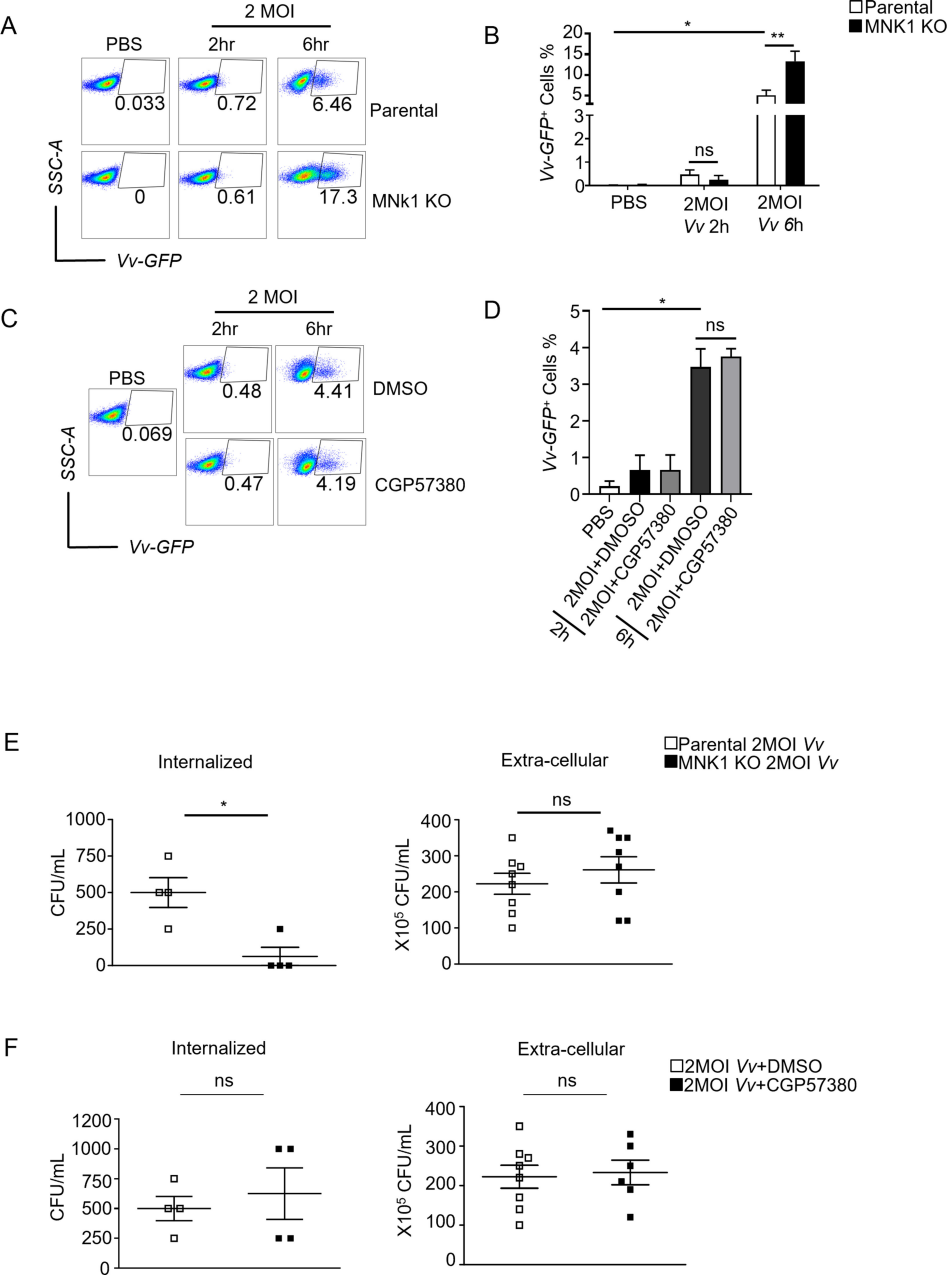
MNK1 hindered the phagocytosis and clearance of *Vv* by macrophages. (**A**) Flow cytometry analysis of GFP-positive cells in MNK1 knockout and parental cells after 2 or 6 h of *Vv*-GFP infection. (**B**) Overlaid histograms show GFP in MNK1 knockout and parental cells after *Vv*-GFP infection. (**C**) Flow cytometry analysis of GFP-positive cells in J774A.1 cells with or without CGP57380 pretreatment before 2 or 6 h of *Vv*-GFP infection. (**D**) Overlaid histograms show GFP in J774A.1 cells with or without CGP57380 pretreatment before *Vv*-GFP infection. (**E**) Internalized and extracellular bacteria of viable *Vv* in MNK1 knockout and parental cells after 6 h of infection. (**F**) Internalized and extracellular bacteria of viable *Vv* in J774A.1 cells with or without CGP57380 pretreatment before 6 h of infection. Data shown are representative of at least three experiments. *, *P* < 0.05; **, *P* < 0.01; ***, *P* < 0.001, determined by Student’s *t*-test.

Macrophages are well-known for their crucial role in the initial defense against pathogens, and phagocytosis is a key process by which macrophages eliminate invading microorganisms. To investigate the involvement of MNK1 in the phagocytic clearance of *Vv* by macrophages, we infected the parental and MNK1^−/−^ J774A.1 cells with *Vv*-GFP for 2 or 6 h. The percentages of *Vv*-GFP-positive J774A.1 cells had slightly increased after 2 h *Vv*-GFP infection. However, there were no differences between parental and MNK1^−/−^ J774A.1 cells after 2 h of *Vv*-GFP infection. But it was interesting that we observed an increased percentage of *Vv*-GFP-positive cells in MNK1^−/−^ cells compared to parental cells after 6-h infection, indicating that a greater number of bacteria were internalized by MNK1^−/−^ cells (Fig. 3A and B). However, using the MNK inhibitor CGP57380 did not significantly affect the percentage of *Vv*-GFP-positive cells following infection (Fig. 3C and D). Meanwhile, it was known to all that *Vv* could cause apoptosis of macrophages (26). To determine whether the increased internalized *Vv* in MNK1^−/−^ cells compared to parental cells was associated with the viability of macrophages, we detected the viability of J774A.1 cells. We observed that MNK1 knockout or the use of the MNK inhibitor did not influence the death of macrophage after 2- or 6-h *Vv-*GFP infection (Fig. S2). Next, we conducted an assessment of the bactericidal activity of J774A.1 cells against the invasion of *Vv* bacteria. We found that MNK1 knockout cells contained a higher number of viable bacteria compared to parental cells after *Vv* infection. Notably, there was no significant difference in extracellular *Vv* between MNK1^−/−^ and parental cells (Fig. 3E). Conversely, treatment with the MNK inhibitor CGP57380 slightly influenced the clearance of both phagocytes internalized and extracellular bacteria in J774A.1 cells following *Vv* infection (Fig. 3F). These results demonstrate that MNK1 negatively regulates the phagocytosis and elimination of *Vv* by J774A.1 cells independent of the MNK-eIF4E signaling pathway.

### MNK1 knockout promotes phagosome acidification of *Vv* infected J774A.1 cells

**Fig 4:**
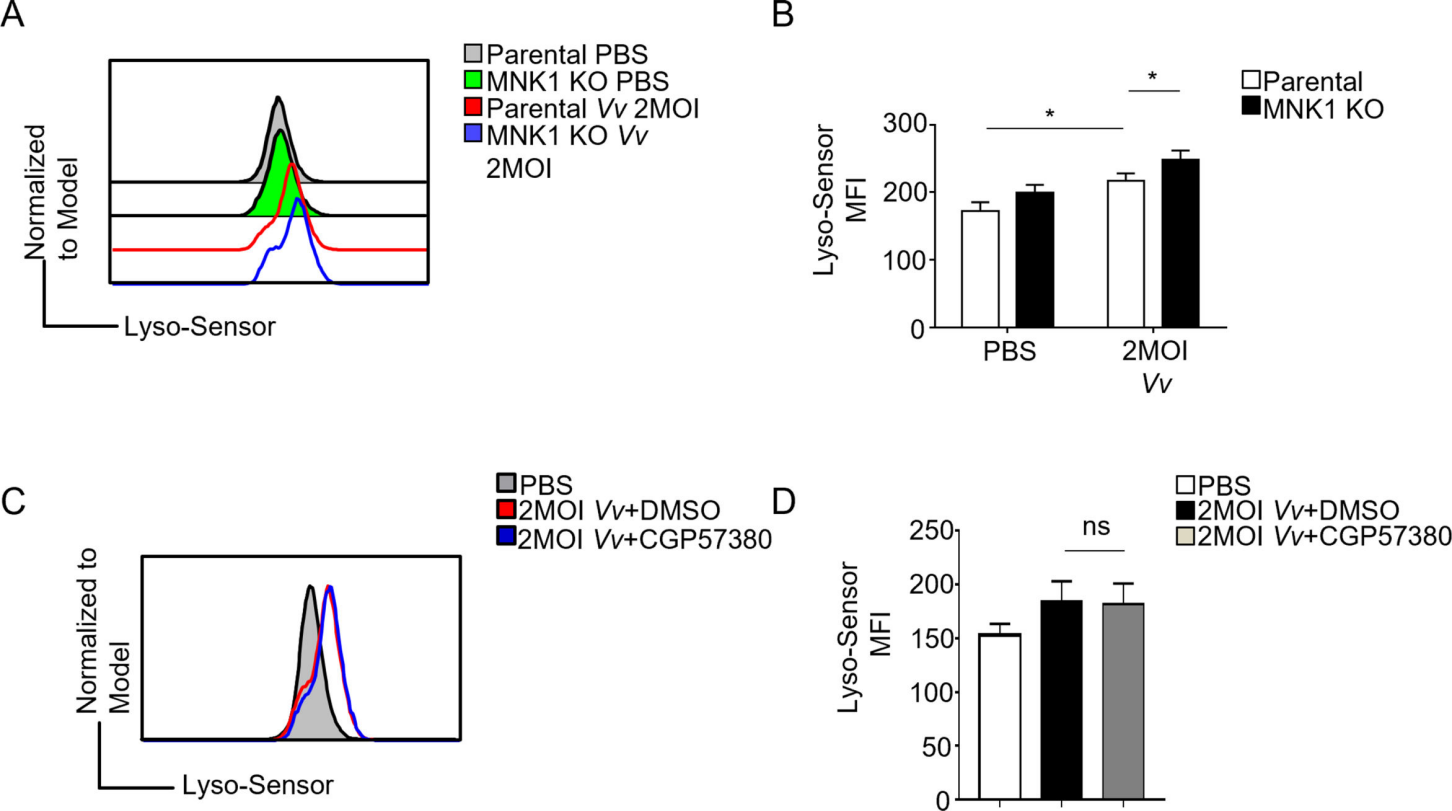
MNK1 impedes the acidification of phagosomes containing *Vv* in macrophages. (**A**) Flow cytometry analysis of LysoSensor intensity in MNK1 knockout and parental cells after 2 MOI *Vv* infection. (**B**) Bar histograms show mean fluorescence intensity (MFI) of LysoSensor in MNK1 knockout and parental cells after 2 MOI *Vv* infection. (**C**) Flow cytometry analysis of LysoSensor intensity in J774A.1 cells with or without CGP57380 pretreatment before 2 MOI *Vv* infection. (**D**) Bar histograms show MFI of LysoSensor in J774A.1 cells with or without CGP57380 pretreatment before 2 MOI *Vv* infection. Data shown are representative of at least three experiments. *, *P* < 0.05; **, *P* < 0.01; ***, *P* < 0.001, determined by Student’s *t*-test.

As it is widely recognized, the bactericidal function of macrophages involves creating an acidic environment and activating pH-sensitive enzymes to degrade internalized bacteria (27). To determine if the enhanced intracellular bacterial killing observed in MNK1-deficient cells results from increased phagosome acidification, we utilized Lysosome Green DND 189 staining. The fluorescence intensity of this stain is negatively correlated with the pH of the phagosome. We assessed the phagosome acidification levels in parental and MNK1^−/−^ J774A.1 cells following *Vv* infection to investigate this. Interestingly, compared to parental cells, we observed stronger acidification in phagosomes containing *Vv* in MNK1-knockout cells (Fig. 4A and B). However, treatment with the MNK inhibitor CGP57380 did not significantly impact phagosome acidity following *Vv* infection (Fig. 4C and D). These findings suggest that the obstruction of phagosome acidification in *Vv*-infected J774A.1 cells by MNK1 is independent of the MNK-eIF4E signaling pathway. It implies that the enhanced intracellular bacterial killing capacity observed upon MNK1 loss may be attributed to the increased acidification of phagosomes.

### MNK1 might hinder the elimination of *Vv* in J774A.1 cells through the MNK1-mTORC1 signaling pathway

**Fig 5:**
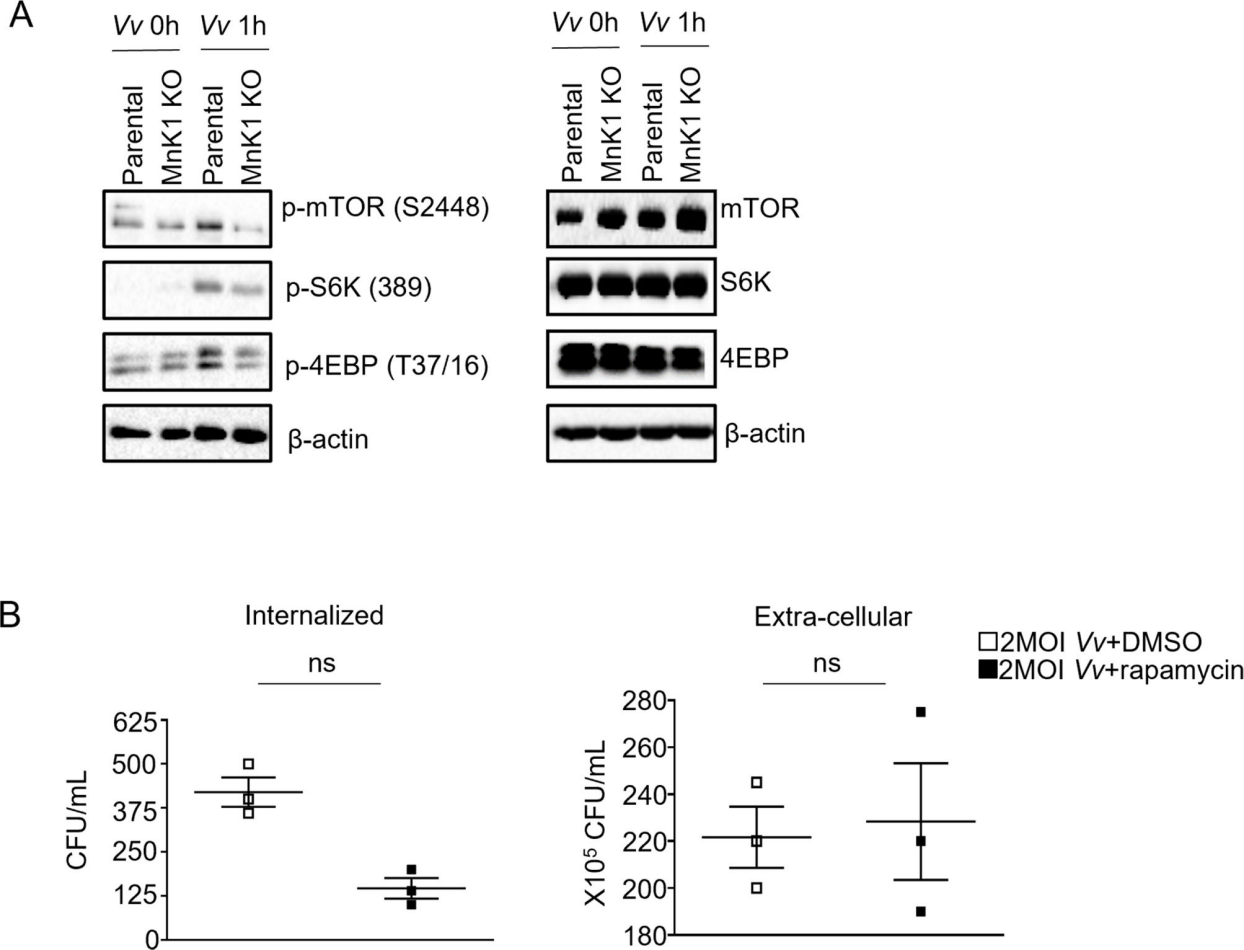
The detrimental impact of MNK1 on the clearance of *Vv* could potentially be attributed to its involvement in the mTORC1 signaling pathway. (**A**) Western blot determined the phosphorylation of mTOR, S6K, and 4EBP in MNK1 knockout and parental cells after 2 MOI *Vv* infection. (**B**) Internalized and extracellular of viable *Vv* in J774A.1 cells with or without rapamycin pretreatment before 6 h of infection. Data shown are representative of at least three experiments. *, *P* < 0.05; **, *P* < 0.01; ***, *P* < 0.001, determined by Student’s *t*-test.

According to previous reports, MNK1 plays a significant role in activating the mTORC1 pathway (28). The absence of MNK1 leads to the inhibition of mTORC1 signaling. Notably, the mTORC1 pathway is closely associated with phagocytosis and clearance of bacteria by macrophages. It has been established that *Vv* can activate the mTORC1 signaling pathway in macrophages (21). We aimed to assess the potential association between MNK1, acting as a negative regulator, and the elimination of *Vv* in J774A.1 cells, with a specific focus on exploring its connection with the mTORC1 signaling pathway. To address this issue, we initially analyzed the activity of the mTORC1 signaling pathway in MNK1-deficient and parental J774A.1 cells following *Vv* infection. As demonstrated in Fig. 5A, upon incubation with *Vv*, MNK1-deficient J774A.1 cells exhibited a significant reduction in the phosphorylation of mTORC1 substrate proteins, including mTOR, S6K, and 4E-BP. Additionally, we observed a decrease in intracellular bacteria burden in J774A.1 cells stimulated by *Vv* that had been pretreated with the mTORC1 inhibitor rapamycin (Fig. 5B). These findings suggest that MNK1 may negatively regulate the elimination of *Vv* in J774A.1 cells through the MNK1-mTORC1 signaling pathway.

## DISCUSSION

MNKs have been found to play significant roles in infection and inflammation. It has been shown that Erk plays a critical role in mediating the production of pro-inflammatory cytokines, which are crucial for controlling the initiation of innate immunity (29). MNKs have been implicated in mediating the production of pro-inflammatory cytokines, such as TNF-α, IL-6, and IL-1β, during infection and inflammation. By controlling mRNA translation through the phosphorylation of eIF4E, MNKs contribute to synthesizing these cytokines, which are crucial for promoting the immune response (22). In experiments using bone marrow-derived macrophages from a Crohn’s-like ileitis mouse model, the pharmacological blockade of MNK resulted in a reduction in the production of TNF-α, IL-6, and MCP-1 (22, 30).

Additionally, inhibition of MNKs negatively regulates IFN-γ and IL-4 in NK cells and blocks IL-1β and IL-8 in Shiga toxin-treated cells. It has been reported that MNK1 mainly regulates the phosphorylation of eIF4E in response to external stimuli such as LPS, while basal levels of eIF4E phosphorylation are controlled by MNK2 (10). Our data confirmed that pharmacological inhibition of MNKs can decrease the production of pro-inflammatory cytokines such as TNF-α and IL-6 in J774A.1 cells treated with LPS, without affecting transcription. Interestingly, the phosphorylation of MNK1 increased while the phosphorylation of eIF4E decreased after pretreatment with CGP57380 before LPS stimulation. This result aligns with a study by Boris V, which found that CGP57380 increased the level of phospho-MNK following LPS stimulation in BMMϕ. However, the underlying reasons for this effect have not been fully elucidated (23).

MNKs have been associated with immune responses against microbial infections. By regulating cytokine production and cellular processes, MNKs contribute to immune defense against microbial pathogens. *Vv* is a Gram-negative bacterium commonly found in warm coastal waters worldwide. Infections with *Vv* can lead to rapidly progressive fatal septicemia and necrotizing wound infections, typically resulting from consuming raw seafood contaminated with the bacterium or exposing an open wound to warm seawater containing *Vv*. Previous studies have shown that *Vv* induces the production of inflammatory cytokines such as IL-1β, IL-6, IL-8, and TNF-α in peripheral blood mononuclear cells from chronic alcohol patients (31). Similarly, *Vv* MO6-24/O LPS has been reported to induce the production of cytokines like IL-1α, IL-6, and TNF-α in rat microglia (32). However, the role of MNKs in regulating the process of *Vv* infection in macrophages remains poorly understood. To investigate this, we first observed that the MNK inhibitor CGP57380 reduced the production of TNF-α and IL-6 in *Vv*-infected J774A.1 macrophage cells without affecting transcription. Additionally, we found that the phosphorylation of MNK1 increased, while the phosphorylation of eIF4E decreased under these conditions. Next, we utilized CRISPR-Cas9 to generate MNK1 knockout J774A.1 cell lines further to explore the role of MNK1 during *Vv* infection. Our results demonstrated that MNK1 was critical in producing TNF-α and IL-6 in J774A.1 cells following *Vv* infection, without any changes in transcription. Furthermore, the MNK1-eIF4E signaling pathway appeared to be involved in the process of *Vv* infection in these cells. These findings suggest that MNK1 may play a role in the defense response of macrophages against *Vv*.

Phagocytic cells, such as neutrophils and macrophages, serve as the next line of defense against pathogens that have breached the epithelial cell barriers. Phagocytosis is a critical antimicrobial mechanism employed by these cells. Our data revealed that the absence of MNK1 enhanced macrophages’ ability to phagocytose and clear *Vv*. It is widely understood that once microbial pathogens are internalized into phagosomes via phagocytosis, these phagosomes progress through a series of steps to form an increasingly acidic compartment, ultimately leading to the elimination of the invading pathogens (33). Interestingly, our findings indicated that the loss of MNK1 promoted phagosome acidification in J774A.1 cells following *Vv* infection without affecting the viability of J774A.1 cells. However, it is important to note that the MNK inhibitor CGP57380 did not impact phagocytosis, bacterial clearance, or phagosome acidification in J774A.1 cells after *Vv* infection. This raises the question of whether MNK1 influences macrophage bactericidal function through pathways other than the MNK1-eIF4E signaling pathway. Further investigation is required to elucidate the alternative mechanisms by which MNK1 may regulate macrophage bactericidal function. MNK1 may interact with other signaling pathways or molecules involved in phagocytosis and phagosome maturation. Understanding these additional pathways could provide valuable insights into the comprehensive role of MNK1 in the functional responses of macrophages during *Vv* infection.

Previous studies have shown that MNK regulates mTORC1 activation by preventing TELO2 binding with mTORC1 (34). Additionally, SLIT2 has been found to promote the killing of bacteria within phagosomes by inhibiting mTORC1 kinase activity in macrophages (35). It has also been reported that AMPK-mediated inhibition of mTORC1 is crucial for selectively targeting bacteria for degradation (36). In the context of *Vv* infection, various signaling pathways, including TLR4 signaling and mTOR signaling, have been implicated (21, 37, 38). In this study, we observed a significant inhibition of mTORC1 signaling in MNK knockout J774A.1 cells infected with *Vv*. Furthermore, our studies have shown that the mTORC1 inhibitor rapamycin promoted *Vv* clearance in J774A.1 cells. Our results suggest that MNK1 may interfere with phagocytosis and macrophage clearance in response to *Vv* infection through the MNK1-mTORC1 signaling pathway rather than the MNK1-eIF4E signaling pathway.

Overall, these findings highlight the importance of the MNK1-mTORC1 pathway in modulating macrophage responses to *Vv* infection. Understanding the intricacies of this signaling pathway could provide valuable insights into the mechanisms underlying the interaction between macrophages and *Vv*, facilitating the development of novel therapeutic interventions for combating this bacterial infection. Further research is needed to fully elucidate the precise molecular mechanisms of how MNK1 regulates mTORC1 signaling during *Vv* infection and its implications for macrophage function.

## MATERIALS AND METHODS

### Bacterial strains and cell culture

J774A.1 cells were purchased from the Cell Bank of Chinese Academy of Science in Shanghai. The cell was cultured in RPMI 1640 containing 10% heat-inactivated fetal bovine serum (Ausvin) and penicillin-streptomycin (50 IU/mL and 50 mg/mL; Beyotime). The China General Microbiological Culture Collection Center provided the *Vv* CGMCC 1.1758 strain. *Vv* grew at 37°C in brain heart infusion (BHI) broth or on the BHI rabbit blood agar plate. After the *Vv* was cultured for 6 h in BHI liquid medium, 100 µL of the *Vv* was diluted 10^5^ times, and 10 µl of the *Vv* was cultured on the plate for 16 h. The total amount of *Vv* was calculated according to the number of bacteria on the plate. Bacteria were washed twice with phosphate-buffered saline (PBS) before use and finally resuspended in PBS. The volume to be added was converted according to the required amount of bacteria for infection.

### Stimulation of J774A.1 cells *in vitro*

We plated 1 × 10^6^ J774A.1 cells in 12-well plates. We then added *Vv* or LPS (*Escherichia coli* O127:B8 from Sigma) to the cells at the indicated MOI or at the indicated concentration pretreated with or without 15-µM CGP57380 for 30 min. We collected supernatants at the indicated time for cytokine quantification. We then exposed the infected cells to the indicated staining and analyzed them for flow cytometry analysis, or cells were lysed in RIPA (Beyotime) with freshly added PMSF (Beyotime) and phosphatase inhibitor cocktails (Sigma) and then subjected for Western blot analysis.

### Phagocytosis of *Vv* by macrophages

For *in vitro* bacterial phagocytosis in macrophages, we incubate J774A.1 cells with 2 MOI of GFP-*Vv* from our laboratory at 37°C for 6 h. Then, we added gentamycin to the culture medium to a final concentration of 100 µg/mL. After incubation at 37°C for 30 min to eliminate the extracellular bacteria, we subjected the cells to flow cytometry to assess GFP levels.

### Phagosome acidification

To measure phagosome acidification, cells were treated with 1-µM LysoSensor Green DND-189 (Thermo Fisher) for 30 min at 37°C. Data were acquired using a BD FACSAria II by excitation at 488 nm.

### Western blot analysis

We subjected cell lysate to Western blot analysis by following the previous protocol (39). Phospho-MNK1 (Thr197/202) Rabbit mAb, MNK1 (C4C1) Rabbit mAb, Phospho-4E-BP1 (Thr37/46) Rabbit mAb, Phospho-mTOR (Ser2448) XP Rabbit mAb, Phospho-p70 S6 Kinase (Thr389) (D5U1O) Rabbit mAb, Phospho-eIF4E (Ser209) Antibody Rabbit, Phospho-p44/42 MAPK (Erk1/2) (Thr202/Tyr204) Antibody, mTOR (7C10) Rabbit mAb, p70 S6 Kinase (49D7) Rabbit mAb, MNK1 (C4C1) Rabbit mAb, and eIF4E (C46H6) Rabbit mAb all came from Cell Signaling Technology. Anti-eIF4EBP1 antibody and anti-ERK1/ERK2 antibody came from Diagbio. Anti-MNK2 (MKNK2) (C-TERMINAL) came from Sigma.

### Generation of GFP-*Vv* strain

We used PCR to amplify the GFP coding sequence from Lv3-pGLV-h1-GFP-pur plasmid (GenePharma) and further cloned it into pUC20 plasmid to generate pUC20-GFP plasmid. Using a BioRad GenePulser Xcell, pUC20-GFP plasmid (100 ng) was introduced into *Vv* by electroporation (40). We identified ampicillin-resistant GFP^+^ *Vv* clones through fluorescence microscopy.

### Generation of MNK1-deficient J774A.1 cell line

We designed a murine MNK1 guide RNA using an online tool (http://crispr.mit.edu). We annealed and cloned oligoes (Forward, 5′-CACC GTC GAA GTC GAG TGT TCC GTG AGG-3′; Reverse 5′-AAAC CCT CAC GGA ACA CTC GAC TTC GAC-3′) corresponding to the guide RNA into pSpCas9 (BB)-2A-GFP (PX458) plasmid (Addgene) to generate PX458-MNK1 following as a previously described protocol (39). We transfected PX458- MNK1 into J774A.1 cells using the QuickShuttle-Superfast Transfection Kit (Biodragon Immunotech), following the manufacturer’s protocol.

### RT-qPCR analysis

We isolated RNA from cells using TRIzol reagents (Omega Bio-tek). We synthesized cDNA using a FastQuant RT Kit (TIANGEN). We performed real-time quantitative PCR (RT-qPCR), as previously described (41) and used ChamQ SYBR qPCR Master Mix (Vazyme) for real-time qPCR. We normalized expression levels of target mRNAs with β-actin and calculated them using the 2-ΔΔCT method. Primers included TNF-α and IL-6 (Table S1).

### Enzyme-linked immunosorbent assay

The levels of TNF-α and IL-6 were measured using commercial ELISA Kits (R&D Systems, USA) according to manufacturer’s instructions.

### Bacterial clearance assay

J774A.1 cells were infected with 2 MOI *Vv* for 6 h and washed with PBS. The supernatant was diluted and plated on blood BHI plates for 12 h. Then, 100-µg/mL gentamicin was added to kill extracellular bacteria. After 30 min, cells were washed twice and treated with 0.1% Triton X-100 and plated on blood BHI plates for 12 h at 37°C for a bacterial count.

### Statistical analysis

Experimental data are expressed as the mean ± SEM. The statistical significance is determined using two-tail Student’s *t*-test. *, *P* < 0.05; **, *P* < 0.01; ***, *P* < 0.001.

## Data Availability

The raw data supporting the conclusions of this article will be made available by the authors, without undue reservation, to any qualiﬁed researcher.

## SUPPLEMENTAL MATERIAL

Supplemental table and figures spectrum.03340-23-s0001.docx Table S1; Fig. S1 and S2.

Supplemental material spectrum.03340-23-s0002.pdf Information for Western blots.

ASM does not own the copyrights to Supplemental Material that may be linked to, or accessed through, an article. The authors have granted ASM a non-exclusive, world-wide license to publish the Supplemental Material files. Please contact the corresponding author directly for reuse.
