# Supplementary material for: The role of MNK1-mTORC1 pathway in modulating macrophage responses to Vibrio vulnificus infection
Source: Microbiol Spectr. 2024 Jul 9;12(8):e03340-23. doi: 10.1128/spectrum.03340-23 (PMC11302032; doi:10.1128/spectrum.03340-23)
Supplement: Supplemental table and figures — Table S1; Fig. S1 and S2. [file spectrum.03340-23-s0001.docx]

**Supplemental Information**

**
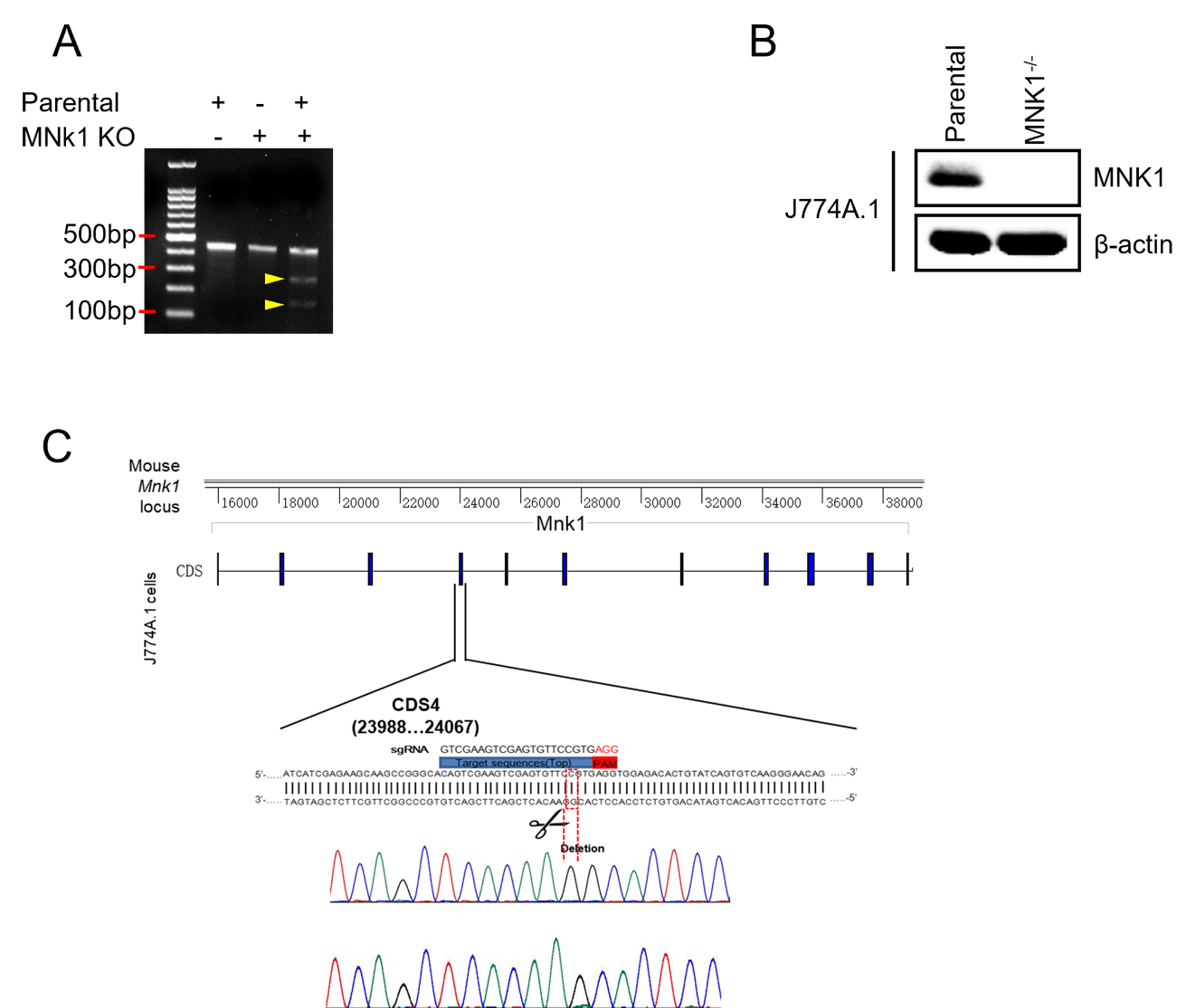
**

**Figure S1.** **Generation of MNK1 knockout cell line using CRISPR/Cas9 gene editing system.** (A) Identification of *MNK1* gene biallelic deletion clones by T7EI digestion assay. (B) Western blot determined the MNK1 expression in MNK1 knockout and parental cell. (C) The schematic of gene editing with CRISPR-Cas9 to generate MNK1 deficient J774A.1 cells.

| 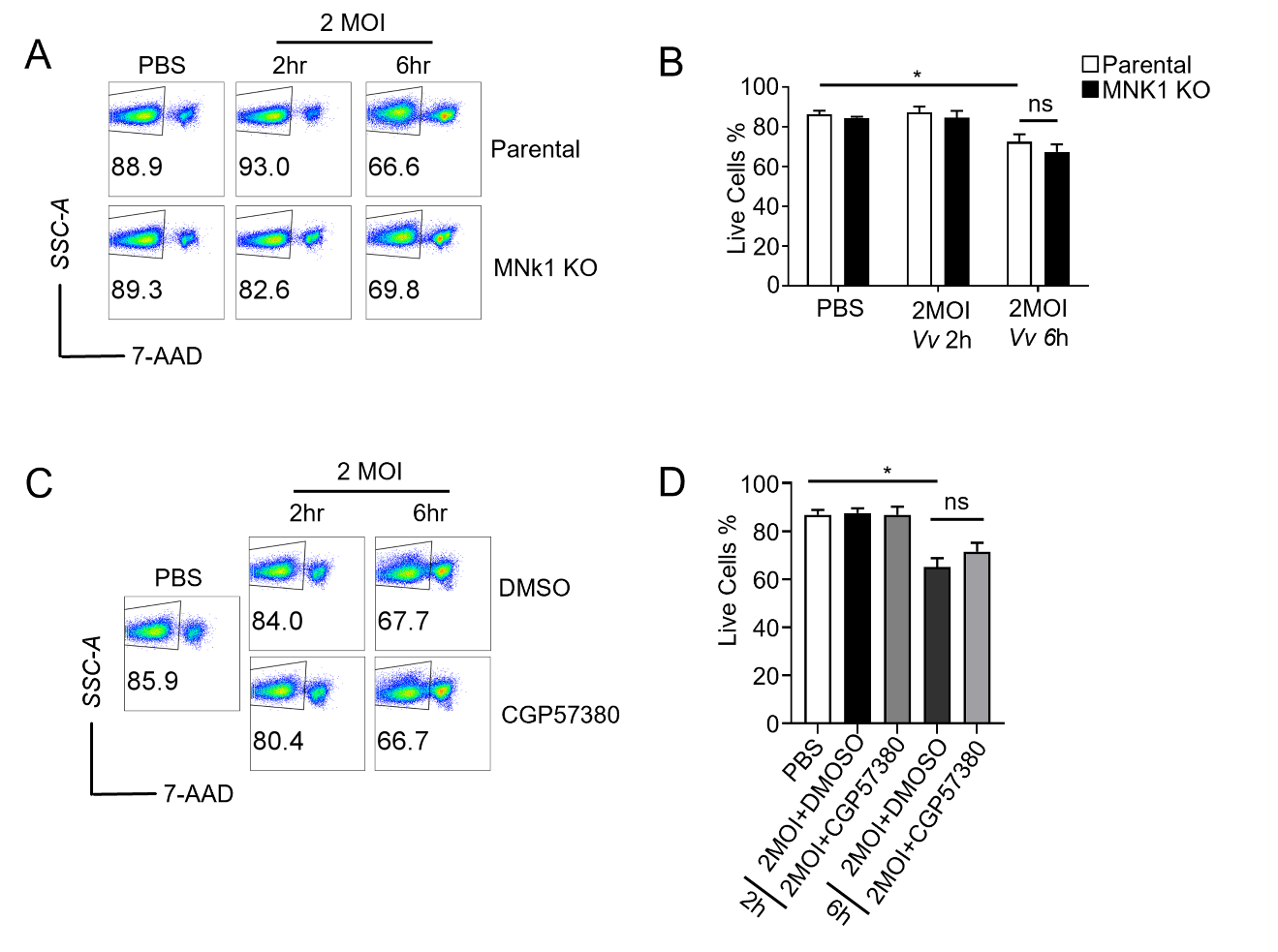  **Figure S2.** **MNK1 knockout or MNK1 inhibitor CGP57380 had no influence in the dead of macrophage after 2h or 6h *Vv*-GFP infection.** (A) Flow cytometry analysis of 7-AAD negative cells in MNK1 knockout and parental cells after 2h or 6h *Vv*-GFP infection. (B) Overlaid histograms show live cells in MNK1 knockout and parental cells after *Vv*-GFP infection. (C) Flow cytometry analysis of 7-AAD negative cells in J774A.1 cells with or without CGP57380 pretreatment before 2h or 6h *Vv*-GFP infection. (D) Overlaid histograms show live cells with or without CGP57380 pretreatment before *Vv*-GFP infection.  **Supplementary Table 1. Primers used in this study** | |
| --- | --- |
| Primer | Sequence (5’–3’) |
| *Tnf-α* F | GGAGA ACCAAGCAACGACAA AATA |
| *Tnf-α* R | TGGGG AACTCTGCAGACTCA AAC |
| *Il-6* F | CGAGACCTCTGGGAAAAAGCT |
| *Il-6* R | GCATACCATAGAGGAATGTGATGTACA |
| *β-actin* F | GGCTGTATTCCCCTCCATCG |
| *β-actin* R | CCAGTTGGTAACAATGCCATGT |
