# Supplementary material for: The role of MNK1-mTORC1 pathway in modulating macrophage responses to Vibrio vulnificus infection
Source: Microbiol Spectr. 2024 Jul 9;12(8):e03340-23. doi: 10.1128/spectrum.03340-23 (PMC11302032; doi:10.1128/spectrum.03340-23)
Supplement: Supplemental material — Information for Western blots. [file spectrum.03340-23-s0002.pdf]

Additional Files and Information for Western blots

Figure S3 Part I (Figure S3A- S3E)

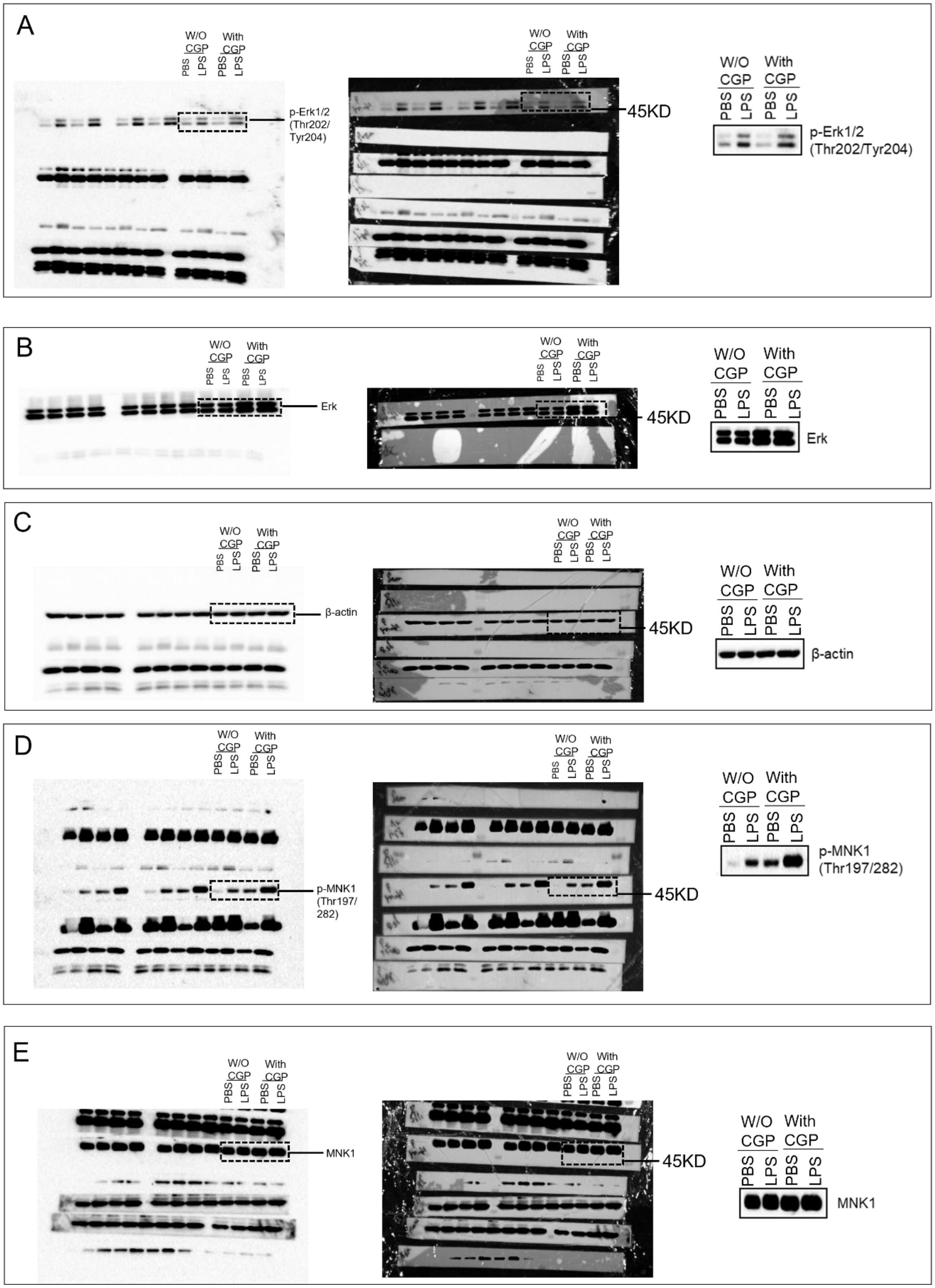

Figure S3 Part II (Figure S3F- S2H)

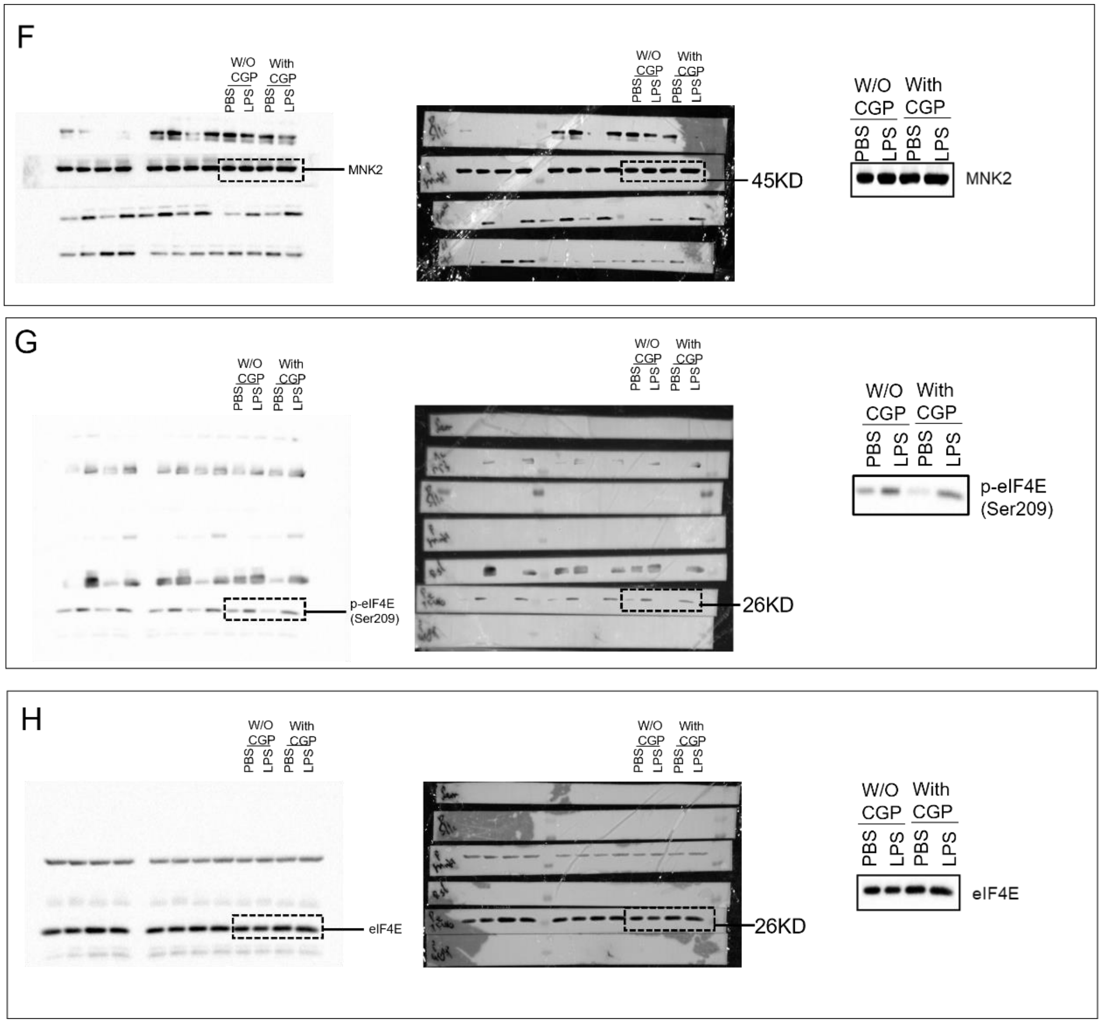

Figure S3. The original blots from Figure 1C. Figure S2A-S2H display the original chemiluminescent and colorimetric blot images related to Figure 1C. In the left panel, the chemiluminescent blot image shows the indicated protein, with the selected image for Figure 1C enclosed in a dashed box. The middle panel consists of an overlay of the original chemiluminescent image with the original colorimetric image for the same protein. Again, the selected image for Figure 1C is enclosed in a dashed box. In the colorimetric blot image, the protein ladder is labeled on the right side of the image. The right panel shows the blot image of the indicated proteins specifically mentioned in Figure 1C.

Figure S4 Part I (Figure S4A- S4E)

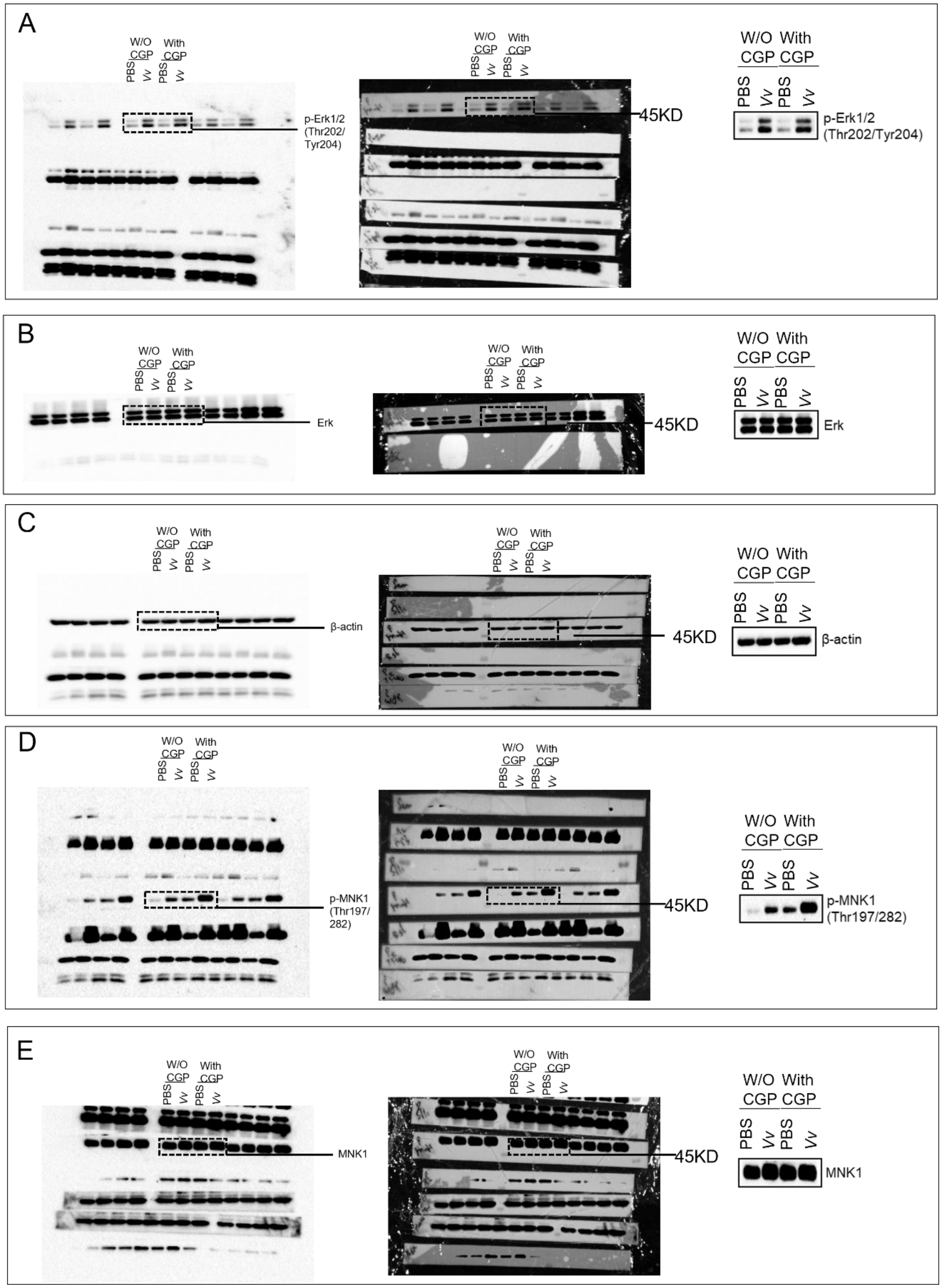

Figure S4 Part II (Figure S4F- S4H)

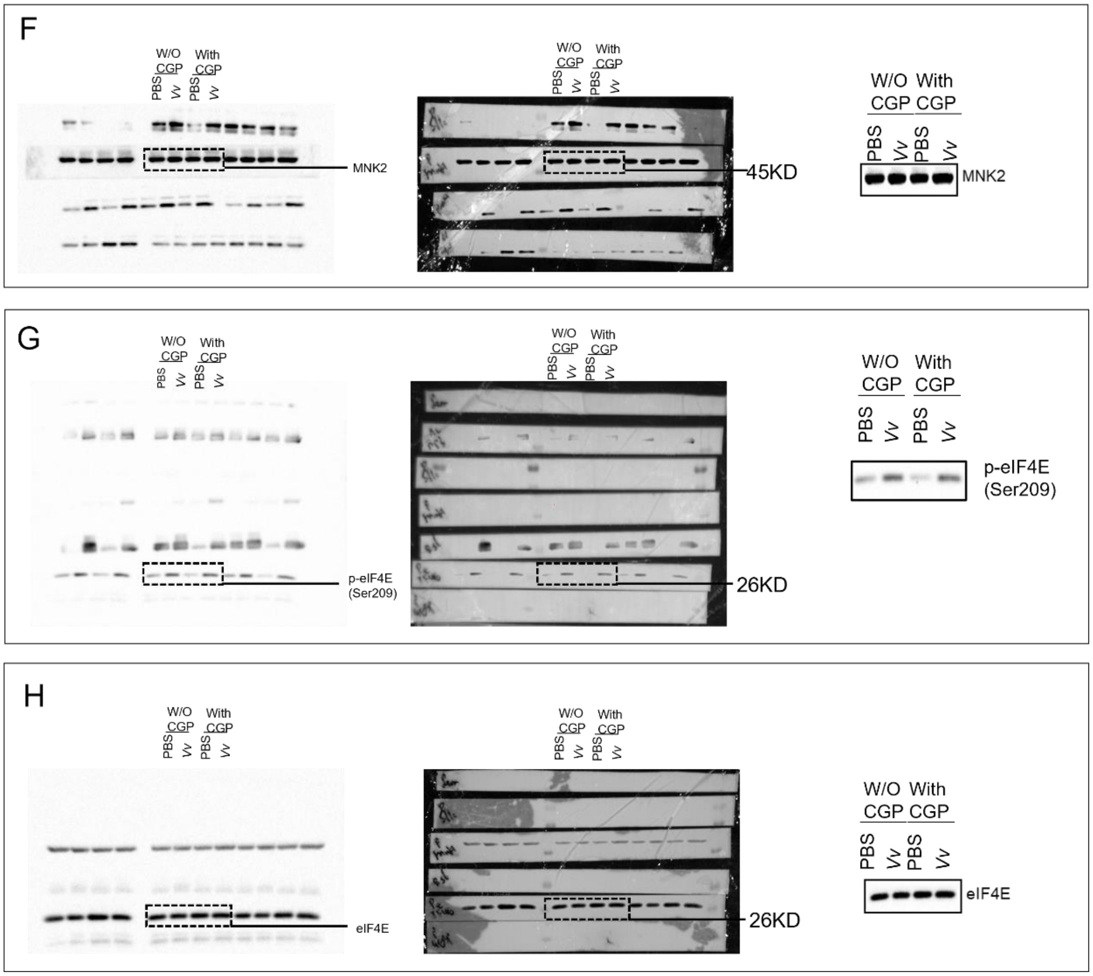

Figure S4. The original blots from Figure 1F. Figure S3A-S3H display the original chemiluminescent and colorimetric blot images related to Figure 1F. In the left panel, the chemiluminescent blot image shows the indicated protein, with the selected image for Figure 1F enclosed in a dashed box. The middle panel consists of an overlay of the original chemiluminescent image with the original colorimetric image for the same protein. Again, the selected image for Figure 1F is enclosed in a dashed box. In the colorimetric blot image, the protein ladder is labeled on the right side of the image. The right panel shows the blot image of the indicated proteins specifically mentioned in Figure 1F.

Figure S5 Part I (Figure S5A- S5D)

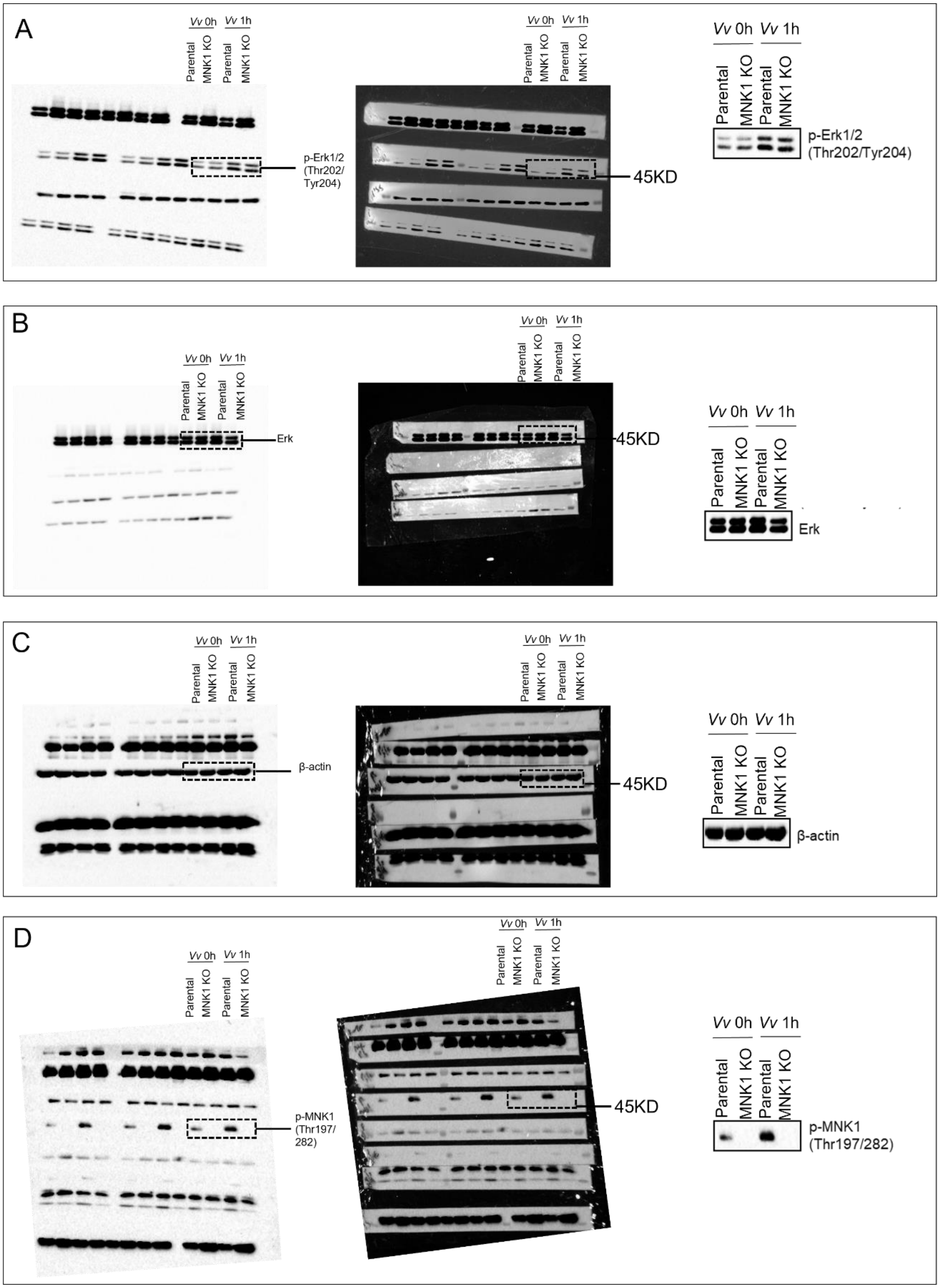

Figure S5 Part II (Figure S5E- S5H)

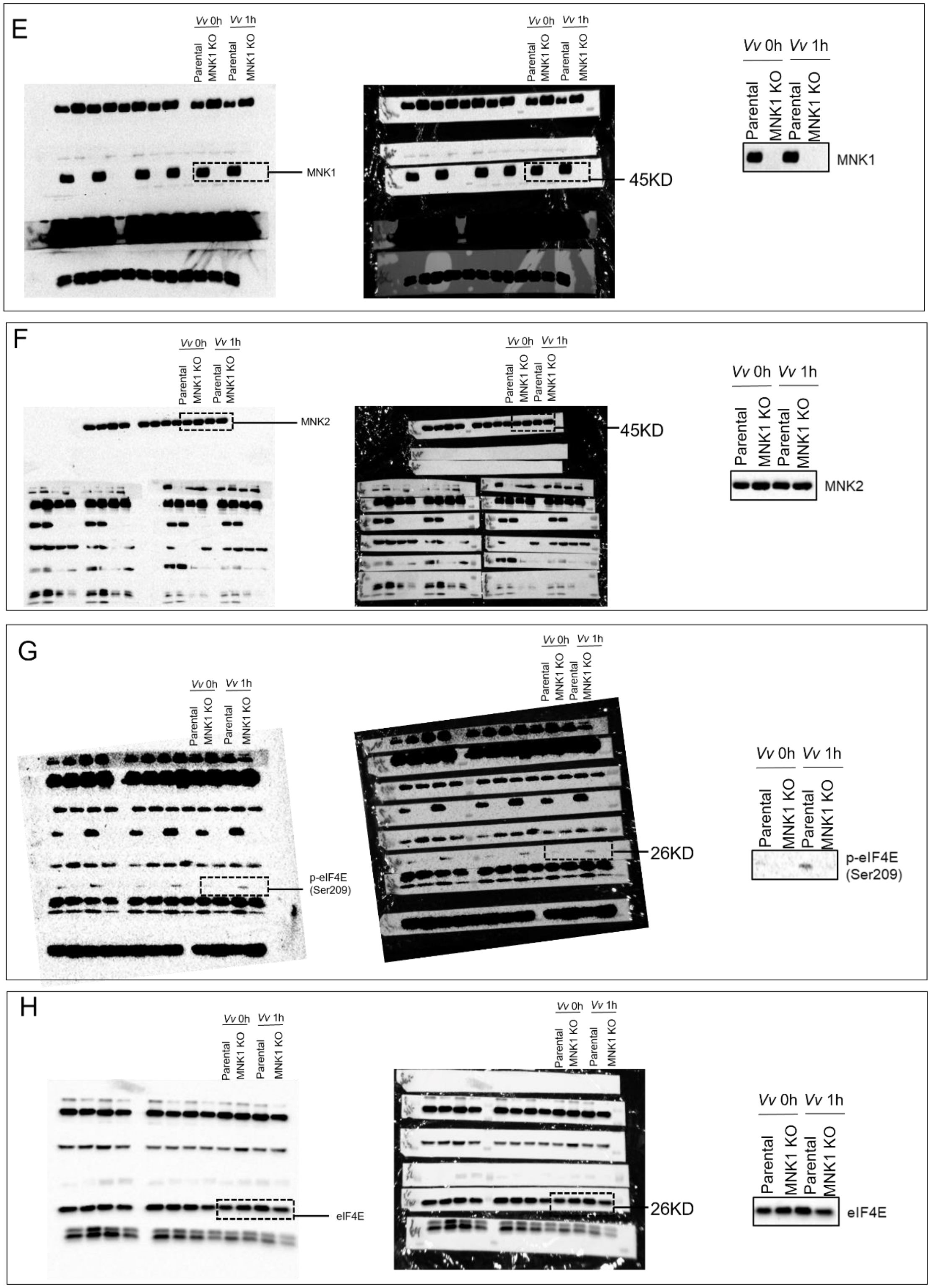

Figure S5. The original blots from Figure 2C. Figure S4A-S4H display the original chemiluminescent and colorimetric blot images related to Figure 2C. In the left panel, the chemiluminescent blot image shows the indicated protein, with the

selected image for Figure 2C enclosed in a dashed box. The middle panel consists of an overlay of the original chemiluminescent image with the original colorimetric image for the same protein. Again, the selected image for Figure 2C is enclosed in a dashed box. In the colorimetric blot image, the protein ladder is labeled on the right side of the image. The right panel shows the blot image of the indicated proteins specifically mentioned in Figure 2C.

Figure S6 Part I (Figure S6A- S6C)

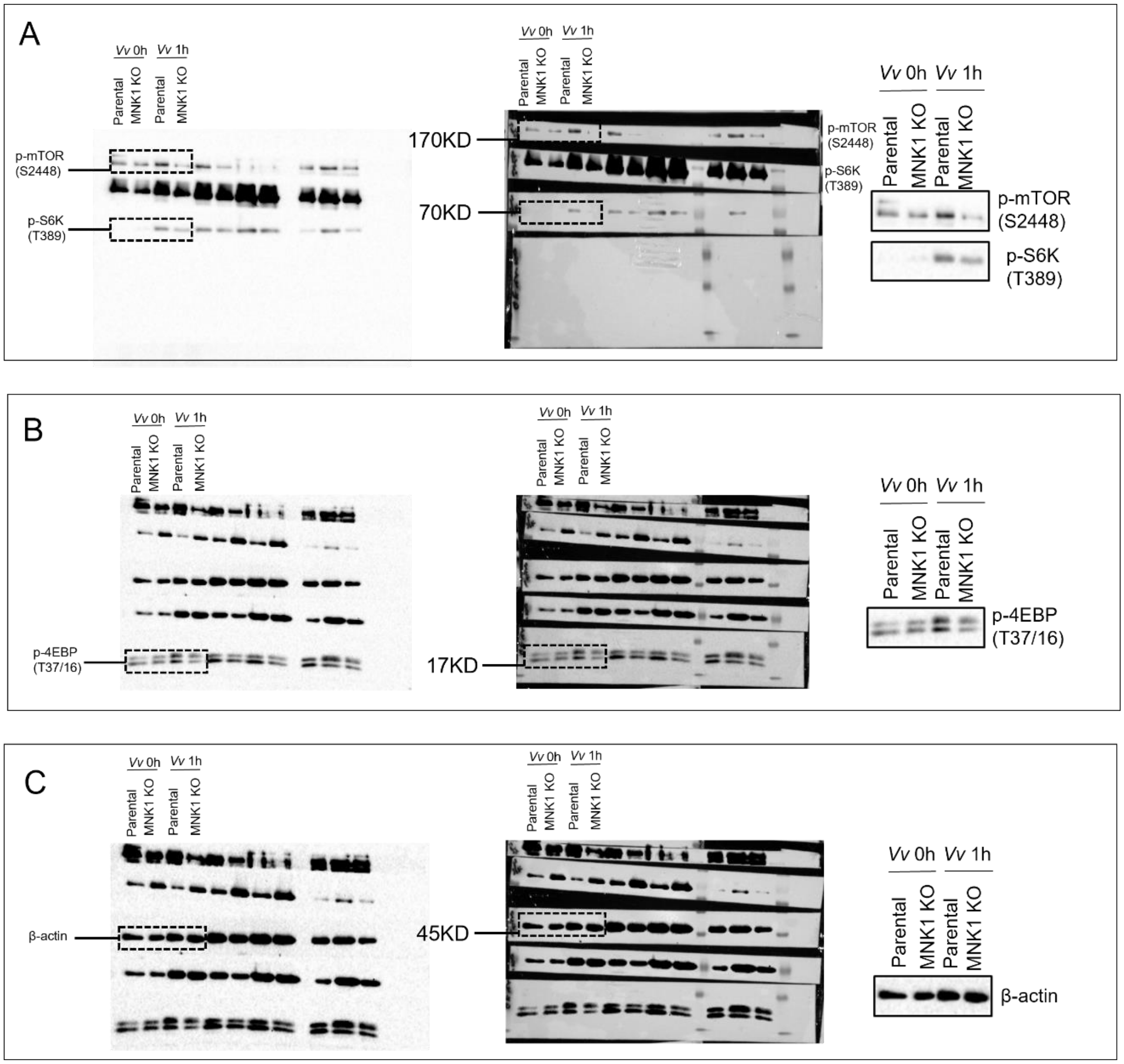

Figure S6 Part II (Figure S6D- S6G)

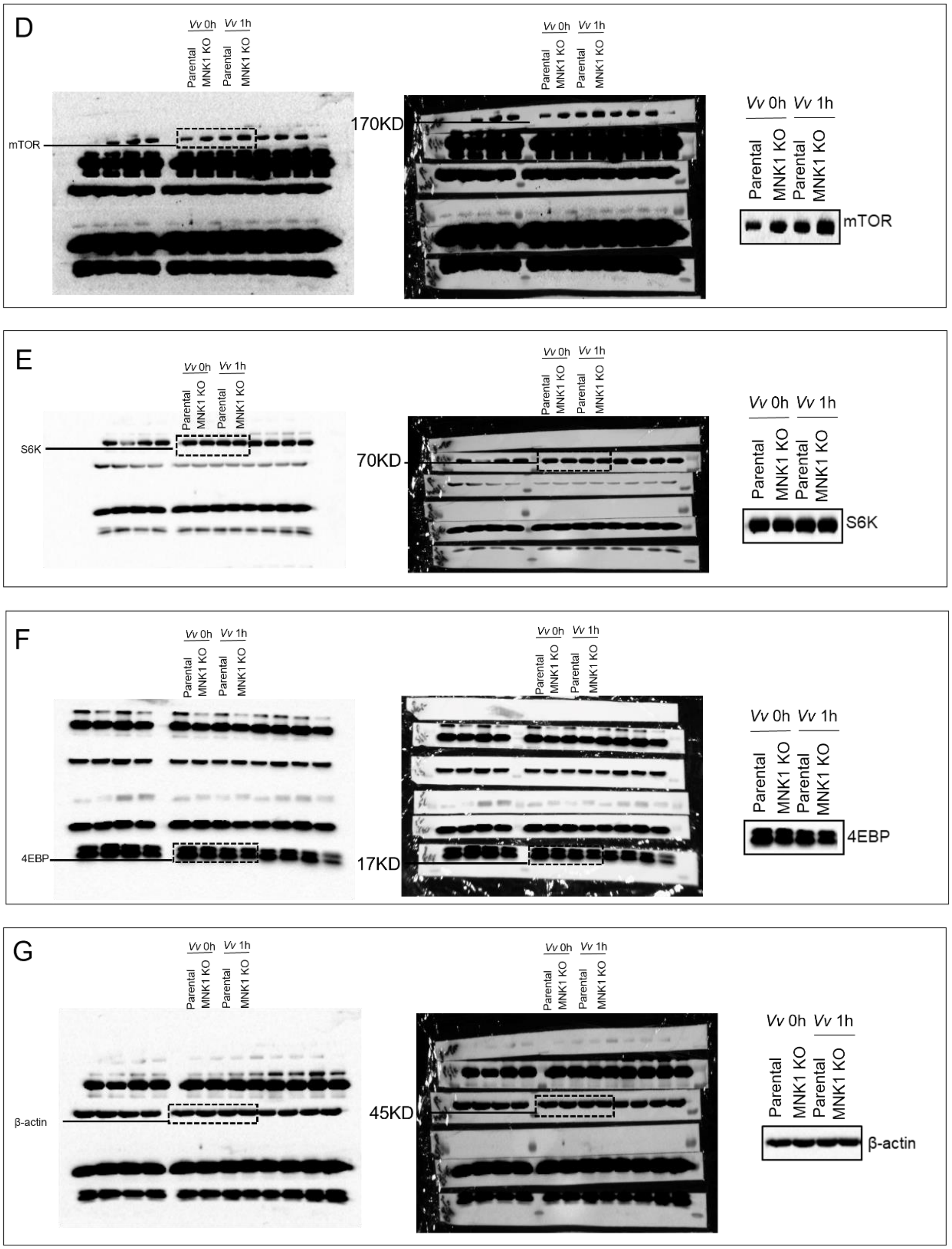

Figure S6. The original blots from Figure 5. Figure S5A-S5G display the original chemiluminescent and colorimetric blot images related to Figure 5A. In the left panel, the chemiluminescent blot image shows the indicated protein, with the selected image for Figure 5A enclosed in a dashed box. The middle panel consists of an overlay of the original chemiluminescent image with the original colorimetric

image for the same protein. Again, the selected image for Figure 5A is enclosed in a dashed box. In the colorimetric blot image, the protein ladder is labeled on the right side of the image. The right panel shows the blot image of the indicated proteins specifically mentioned in Figure 5A.

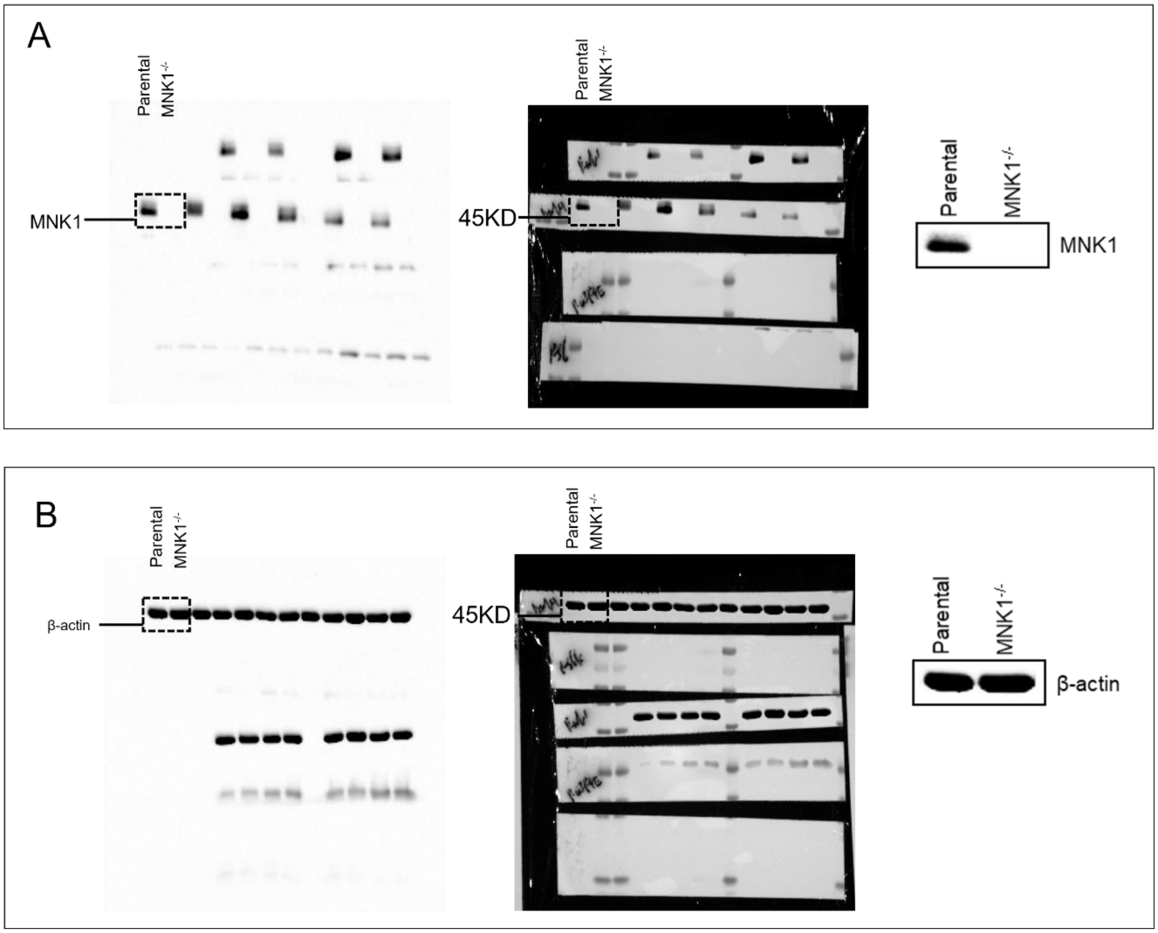

Figure S7. The original blots from Figure S1B. Figure S6A and S6B display the original chemiluminescent and colorimetric blot images related to Figure S1B. In the left panel, the chemiluminescent blot image shows the indicated protein, with the selected image for Figure S1B enclosed in a dashed box. The middle panel consists of an overlay of the original chemiluminescent image with the original colorimetric image for the same protein. Again, the selected image for Figure S1B is enclosed in a dashed box. In the colorimetric blot image, the protein ladder is labeled on the right side of the image. The right panel shows the blot image of the indicated proteins specifically mentioned in Figure S1B.
